# Universal Newborn Screening for Glucose-6-Phosphate Dehydrogenase (G6PD) Deficiency in a Safety-Net Well-Baby Nursery: A Quality Improvement Initiative

**DOI:** 10.3390/children13091219

**Published:** 2026-09-09

**Authors:** Sheetal Sriraman, Charlotte Banayan, Sana Usmani, Saema Khandakar, Ivan Hand

**Affiliations:** 1 Department of Pediatrics, Division of Neonatal and Perinatal Medicine, Columbia University Irving Medical Center, New York, NY 10032, USA; 2 Department of Pediatrics, Division of Neonatal and Perinatal Medicine, NewYork-Presbyterian Morgan Stanley Children’s Hospital, New York, NY 10032, USA; 3 Department of Pediatrics, Division of Gastroenterology, Children’s Hospital of Los Angeles, Los Angeles, CA 90027, USA; 4 Department of Pediatrics, NYC Health + Hospitals/Kings County, Brooklyn, NY 11203, USA

**Keywords:** neonatology, newborn medicine, hematology, bilirubin metabolism, kernicterus, quality improvement, screening, newborn screening, glucose-6-phosphate dehydrogenase

## Abstract

**Highlights:**

**What are the main findings?**

- Universal G6PD deficiency screening in a safety-net well-baby nursery was successfully implemented and sustained, rising from 0% to a mean of 81.1% of infants screened through quality improvement methodology.
- Nearly 1 in 11 screened neonates (9.0%) tested positive for G6PD deficiency, and these infants had higher bilirubin levels and required more bilirubin testing.

**What are the implications of the main findings?**

- Universal newborn G6PD screening can be built into existing nursery workflows, offering an equitable alternative to risk-based approaches.
- Identifying neonates who screen positive for G6PD deficiency at birth allows closer bilirubin monitoring and follow-up to be targeted to the infants at greatest risk of severe hyperbilirubinemia and kernicterus, especially in populations most vulnerable to care gaps.

**Abstract:**

**Background**: G6PD deficiency is among the leading causes of neonatal hyperbilirubinemia and kernicterus. Following a 2022 New York State Department of Health recommendation to test high-risk neonates, we implemented universal G6PD deficiency screening in the well-baby nursery. **Objectives**: We aimed to increase the proportion of infants screened from 0% to more than 75% within 6 months and to describe the prevalence of G6PD deficiency and the early outcomes of affected neonates. **Methods**: This quality improvement (QI) initiative, guided by the Model for Improvement, included a cross-sectional analysis of screening yield and early neonatal outcomes. A statistical process control p-chart tracked monthly screening. Outcomes were compared between screen-positive and screen-negative infants using the Fisher exact test. **Results**: Screening rose from 0% to a sustained mean of 81.1%, exceeding the 75% aim. Of 580 screened neonates, 52 (9.0%) screened positive for G6PD deficiency. Screen-positive infants were more likely than screen-negative infants to undergo repeat serum bilirubin testing (38.5% vs. 19.3%; *p* = 0.002) and to reach a peak bilirubin above 10 mg/dL (30.8% vs. 10.2%; *p* < 0.001). A higher rate of readmission for phototherapy was also observed (5.8% vs. 0.9%; *p* = 0.028), though based on few events. Phototherapy during the birth hospitalization, IVIG, and exchange transfusion did not differ. **Conclusions**: Universal G6PD screening was feasibly implemented and sustained in a high-risk well-baby nursery through routine workflow changes, without additional phlebotomy. Nearly 1 in 11 screened neonates tested positive for G6PD deficiency.

## 1. Introduction

Glucose-6-phosphate dehydrogenase (G6PD) is an enzyme essential for protecting red blood cells from oxidative damage. G6PD deficiency, an X-linked disorder and the most common enzyme deficiency globally, affects populations of African, Mediterranean, Middle Eastern, and Asian descent [1]. In neonates, G6PD deficiency is a significant cause of severe neonatal jaundice, potentially leading to kernicterus and permanent neurological damage if untreated [2]. For these reasons, pediatric government bodies have recommended G6PD deficiency testing at the discretion of the care provider based on the infant’s risk [3,4]. Routine screening for G6PD deficiency has already been implemented in countries like Singapore and Greece over two decades ago, as part of their newborn screening programs [5,6].

In the United States, G6PD deficiency is not part of the Recommended Uniform Screening Panel (RUSP) [7]. However, states like Pennsylvania and Washington, D.C., include it in their newborn screening programs [8]. In June 2022, New York State amended its newborn screening statute to recommend G6PD deficiency testing for neonates with specific risk factors, including persistent jaundice, hemolytic anemia, or familial, racial, or ethnic predisposition [9]. This amendment did not mandate universal screening, placing the responsibility for testing on clinicians based on individual patient risk [8]. Recognizing the diverse and high-risk population served at our institution, a universal G6PD deficiency screening was implemented.

The primary aim of this initiative was to increase the proportion of infants screened for G6PD deficiency from 0% to more than 75% within 6 months. Secondary aims were to determine the proportion of screened infants who tested positive for G6PD deficiency and to compare the early neonatal outcomes of infants with and without G6PD deficiency.

## 2. Methods

### 2.1. Setting

This study was carried out in the well-baby nursery at a public hospital in New York City that is affiliated with an academic institution. This hospital serves a predominantly African American and African Caribbean population (approximately 80%) [10]. The hospital has approximately 1300–1400 live births a year, and the nursery has an average daily census of 10 infants [11]. The nursery is staffed by an interdisciplinary team of attending physicians, resident physicians, nurses, nursing assistants, and social workers.

Standard practice for all newborns admitted to this unit is to perform a serum total and direct bilirubin level and state-mandated newborn screening test at approximately 30 h of life (usually between 24–36 h of life). Infants who are at risk for hyperbilirubinemia, such as infants who are ABO incompatible or Coombs-positive, may receive more frequent assessment of serum bilirubin levels.

### 2.2. Policy Change and G6PD Deficiency Screening

In 2022, New York State amended its statute for newborn screening to include testing for G6PD deficiency, stating that it is the duty of those caring for neonates to test neonates who qualify based on several risk factors such as persistent jaundice or hemolytic anemia, admission to the hospital for jaundice after birth, or a parent with familial, racial, or ethnic risk for G6PD deficiency (e.g., African, Asian, Mediterranean, or Middle Eastern ancestry) [9].

An expert council comprising neonatologists, pediatricians, and hematologists convened following the publication of the amended statute to evaluate its impact on the patient population served by our hospital. Given that the institution primarily serves a population of African American and African Caribbean patients, the council decided to implement universal G6PD deficiency screening for all neonates admitted to the well-baby nursery. This new screening protocol was introduced as a pilot program in the well-baby nursery, with plans for future expansion to the neonatal intensive care unit (NICU) following the initial implementation phase. The council determined that the Model for Improvement framework was the most suitable approach for implementing universal G6PD deficiency screening in the well-baby nursery.

### 2.3. G6PD Testing and Logistics

G6PD deficiency screening was performed using a quantitative spectrophotometric assay, which required less than 0.2 mL of blood collected in an Acid Citrate Dextrose (ACD) microtainer or a lithium heparin microtainer. Since the institution’s laboratory could not process quantitative G6PD levels, the tests were sent to an external laboratory. The external laboratory is certified under the Clinical Laboratory Improvement Amendments (CLIA) and accredited by the College of American Pathologists (CAP). Quantitative G6PD activity was measured by spectrophotometry. Blood collection for G6PD deficiency screening occurred at approximately 30 h of life, alongside serum bilirubin testing and the state-mandated newborn screening. No additional blood was needed, as the G6PD quantitative test was performed using the same yellow-top microtainer used for serum bilirubin testing. Parents were appropriately counseled about the G6PD deficiency screening at the time of specimen collection and were informed of their right to refuse the test. Screening was incorporated into routine newborn clinical care under institutional policy; verbal parental notification with the opportunity to decline was provided, so separate written informed consent for screening was not required. All questions were addressed by the frontline team during this process.

### 2.4. Results of G6PD Deficiency Screening and Follow-Up

G6PD deficiency screening results were typically available within 5 to 7 days, often after most infants had been discharged from the nursery. All clinical decisions during the nursery admission were made blinded to G6PD status. Consequently, the responsibility for following up on G6PD deficiency screening results fell to the infant’s primary care provider. Quantitative G6PD activity was reported by the reference laboratory in U/g Hb. Because published newborn cutoffs vary and were derived from populations different from ours, we additionally derived population-specific thresholds from our own cohort using the adjusted male median (AMM) method. Deficiency was defined a priori as <30% of the AMM and intermediate activity as 30–80% of the AMM. Infants identified with low quantitative G6PD levels were referred to a dedicated pediatric hematology clinic, which was established specifically to address the needs of infants flagged by the screening. At the pediatric hematology clinic, repeat testing was performed to confirm the diagnosis. Parents and families received detailed counseling and were provided with educational resources to help them navigate the diagnosis. Additionally, genetic counseling was offered to families interested in further understanding the condition and its implications.

### 2.5. Study Design

This study was conducted in two parts: a quality improvement initiative using the Model for Improvement to implement universal G6PD screening and a cross-sectional analysis of screening yield and the outcomes of infants with and without G6PD deficiency. The quality improvement component (Part 1) was implemented and reported in accordance with the Standards for Quality Improvement Reporting Excellence (SQUIRE 2.0) guidelines and comprised the G6PD deficiency screening implementation. The observational component (Part 2) was a cross-sectional evaluation of the results of G6PD deficiency screening, the distribution of G6PD activity, and early neonatal outcomes among screened infants, and was reported in accordance with the Strengthening the Reporting of Observational Studies in Epidemiology (STROBE) recommendations.

#### 2.5.1. Part 1: Quality Improvement Implementation of Universal G6PD Screening

The QI team included attending and resident physicians. The interdisciplinary team of stakeholders included pediatric attending physicians, hematologists, neonatologists, resident physicians, nurses, nursing assistants, and social workers. This interdisciplinary team collaborated closely before and during the testing phase, and met monthly to discuss ongoing progress and troubleshoot barriers.

Baseline data were collected between July 2022 and August 2022. Interventions began in September 2022, and the intervention phase continued from September 2022 to December 2022, followed by which there was a sustainability phase from January 2023 to April 2023.

##### Improvement Interventions

**Figure 1:**
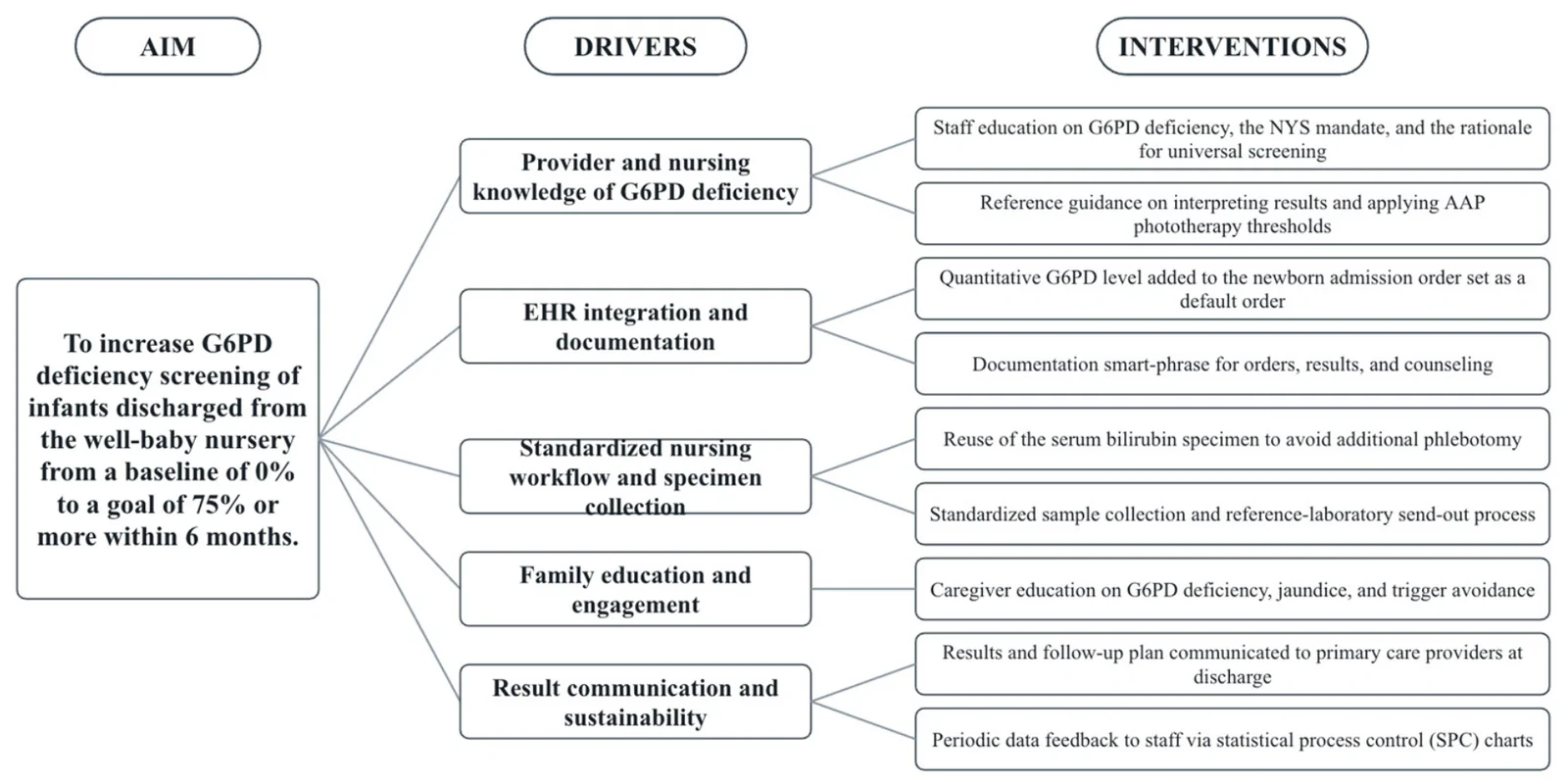
Key Driver Diagram.

A key driver diagram that was collaboratively developed by the study team and the interdisciplinary team was used to guide improvement efforts (Figure 1). Interventions began in September 2022 and included the following:

(a) Staff training: Training sessions were conducted to familiarize residents and nursing staff with the G6PD deficiency screening process. Residents were trained to include quantitative G6PD deficiency testing as part of their admission orders, communicate effectively with parents and families about the purpose and importance of G6PD deficiency screening, and document relevant information in discharge summaries. Nursing staff received instruction on the proper techniques for collecting and sending the specimen, the appropriate destination for the specimen, and key points to include when counselling parents and families.
(b) Primary Care Provider Education: Since primary care providers were responsible for following up on G6PD deficiency screening results for most infants discharged from the well-baby nursery, targeted education was provided. This training included the need for providers to track screening results, counsel parents on the findings, and ensure timely referrals to the pediatric hematology clinic for infants who screened positive for G6PD deficiency.
(c) Availability of Literature and Educational Material: To support the staff in their roles, relevant educational materials were distributed, ensuring that nurses, residents, primary care providers, and other frontline personnel had easy access to resources about G6PD deficiency.
(d) Scripts for Discussing G6PD Deficiency Screening with Parents: Scripts were developed to standardize discussions about G6PD deficiency screening with parents, enabling clear and consistent communication.
(e) Weekly Check-ins with Frontline Staff: Regular weekly check-ins with frontline staff created an opportunity to gather feedback, address challenges, and troubleshoot barriers.
(f) Monthly G6PD Deficiency Screening Huddles: Monthly huddles with the interdisciplinary team of stakeholders and the study team provided a platform to review progress, share updates, and discuss strategies for optimizing workflow.
(g) Documentation of Screening: Discharge documentation templates were revised to include G6PD deficiency screening status and results, when available, for all infants discharged from the well-baby nursery.
(h) Electronic Health Record (EHR) Optimization: The EHR system was optimized to include the automatic selection of quantitative G6PD levels in the well-baby nursery admission order set to streamline the ordering process and ensure that screening was routinely performed.

The diagram links the overall aim to the primary drivers (provider and nursing knowledge of G6PD deficiency, EHR integration and documentation, standardized nursing workflow and specimen collection, family education and engagement, and results communication and sustainability).

##### Measures

Outcome measures:

The proportion of infants discharged from the well-baby nursery who were screened for G6PD deficiency. An infant was counted as screened when a quantitative G6PD level was ordered, the specimen was collected and sent to the laboratory during the birth hospitalization, and a quantitative result was returned.

Balancing measures:

To capture unintended effects on the care team, we tracked staff-reported workload as a balancing measure: during weekly check-ins, frontline nursing and provider staff were asked whether the added screening had increased their workload.

Because G6PD screening reused the specimen already obtained for serum bilirubin and required no additional venipuncture or blood volume, direct harm from the intervention itself was not anticipated.

#### 2.5.2. Part 2: Evaluation of the Proportion of Infants with G6PD Deficiency on Initial Screening and Their Outcomes

**Figure 2:**
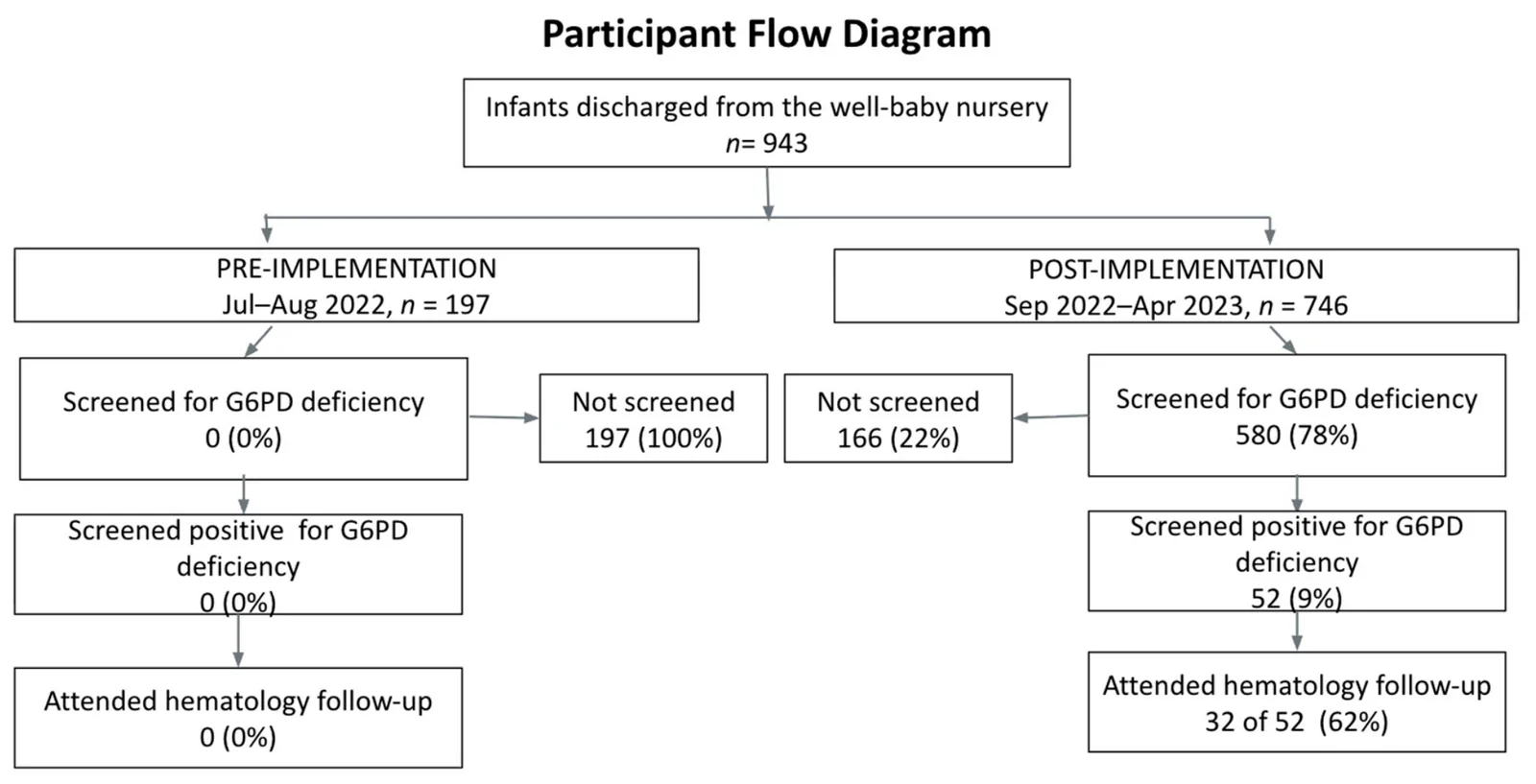
Participant flow diagram.

After implementing the G6PD deficiency screening program, a retrospective chart review was conducted to assess the proportion of infants diagnosed with G6PD deficiency based on screening results. The review also analyzed the demographics of infants who screened positive for G6PD deficiency and compared the outcomes of infants with and without G6PD deficiency. Outcomes included in the analysis are the need for repeat serum bilirubin testing, the need for phototherapy, the need for IVIG, and the need for exchange transfusion. Repeat serum bilirubin testing was defined as any serum bilirubin measurement obtained after the routine screening of bilirubin during the birth hospitalization. Hospitalization for hyperbilirubinemia was defined as readmission for phototherapy within 30 days of discharge. Peak serum bilirubin was defined as the highest serum bilirubin level recorded at any point during the birth hospitalization and was analyzed at thresholds of greater than 10 and greater than 15 mg/dL. All infants were discharged from the well-baby nursery at New York City Health and Hospitals, Kings County. The study was time-bound and included all infants discharged from the well-baby nursery from July 2022 until April 2023. Eligibility was determined through chart review. Eligible infants were all neonates discharged from the well-baby nursery during the study period. Infants who were not discharged from the well-baby nursery, including those admitted directly to or transferred to the neonatal intensive care unit, were not included. No additional exclusions were applied on the basis of gestational age, birth weight, or congenital anomalies. Infants with a G6PD deficiency test order who were discharged before specimen collection, whose specimens were inadequate, or whose parents declined testing were classified as non-screened. The participant flow diagram is summarized in Figure 2.

Ethical considerations:

This study was evaluated by the Institutional Review Board (IRB) (ID: 23-12-501-202). The quality improvement portion was considered to be not research and was considered exempt. The retrospective cross-sectional analysis of screening and neonatal outcomes was conducted under this same Institutional Review Board determination, which waived the requirement for informed consent.

Analysis:

A statistical process control (SPC) p-chart was created in Excel (Microsoft Corporation Version 2605) using QI macros to track the monthly proportion of infants screened for G6PD deficiency. Each data point in the SPC chart represented the proportion of infants tested for G6PD deficiency during a given month. The numerator was the number of infants tested for G6PD deficiency, and the denominator was the total number of infants discharged from the well-baby nursery that month. A change on the SPC chart was considered non-random and significant if it met the criteria outlined by the Institute for Healthcare Improvement [12]. No G6PD screening existed in the nursery before this initiative, so the two baseline months (July and August 2022) had zero infants who received G6PD deficiency screening. Given that the baseline levels were zero and the baseline months were not just low-prevalence epochs, an SPC chart was thought to be most appropriate.

Descriptive statistics were utilized to report the number and proportion of infants who screened positive for G6PD deficiency, as well as the demographics of infants included in the study. Categorical outcomes were compared between infants who screened positive for G6PD deficiency and those who did not using the Fisher exact test. The Fisher exact test was applied uniformly to all categorical comparisons. A *p*-value of less than 0.05 was considered statistically significant. Because multiple outcomes were compared and no adjustment for multiple comparisons was performed, these analyses should be regarded as exploratory and hypothesis-generating. Population-specific thresholds for G6PD deficiency were derived from our own cohort using the adjusted male median (AMM) method [13]. To derive the adjusted male median, the initial median was calculated from all measured male values, after which presumptive deficient outliers (<10% of the overall male median) were excluded and the median was recalculated using the remaining values. Given that males are hemizygous for G6PD, the resulting adjusted male median was considered an estimate of 100% normal enzyme activity in the screened population and was used as the reference for the prespecified deficiency and intermediate-activity thresholds. Deficiency was defined a priori as <30% of the AMM and intermediate activity as 30–80% of the AMM. Infants whose specimens returned a normal or high result without a numeric value were excluded from distributional analyses but retained in all prevalence denominators. Statistical analyses were conducted using JASP (Version 0.97.0) [14].

## 3. Results

Part 1: Uptake and Sustainability of G6PD Deficiency Screening (Quality Improvement Component)

**Figure 3:**
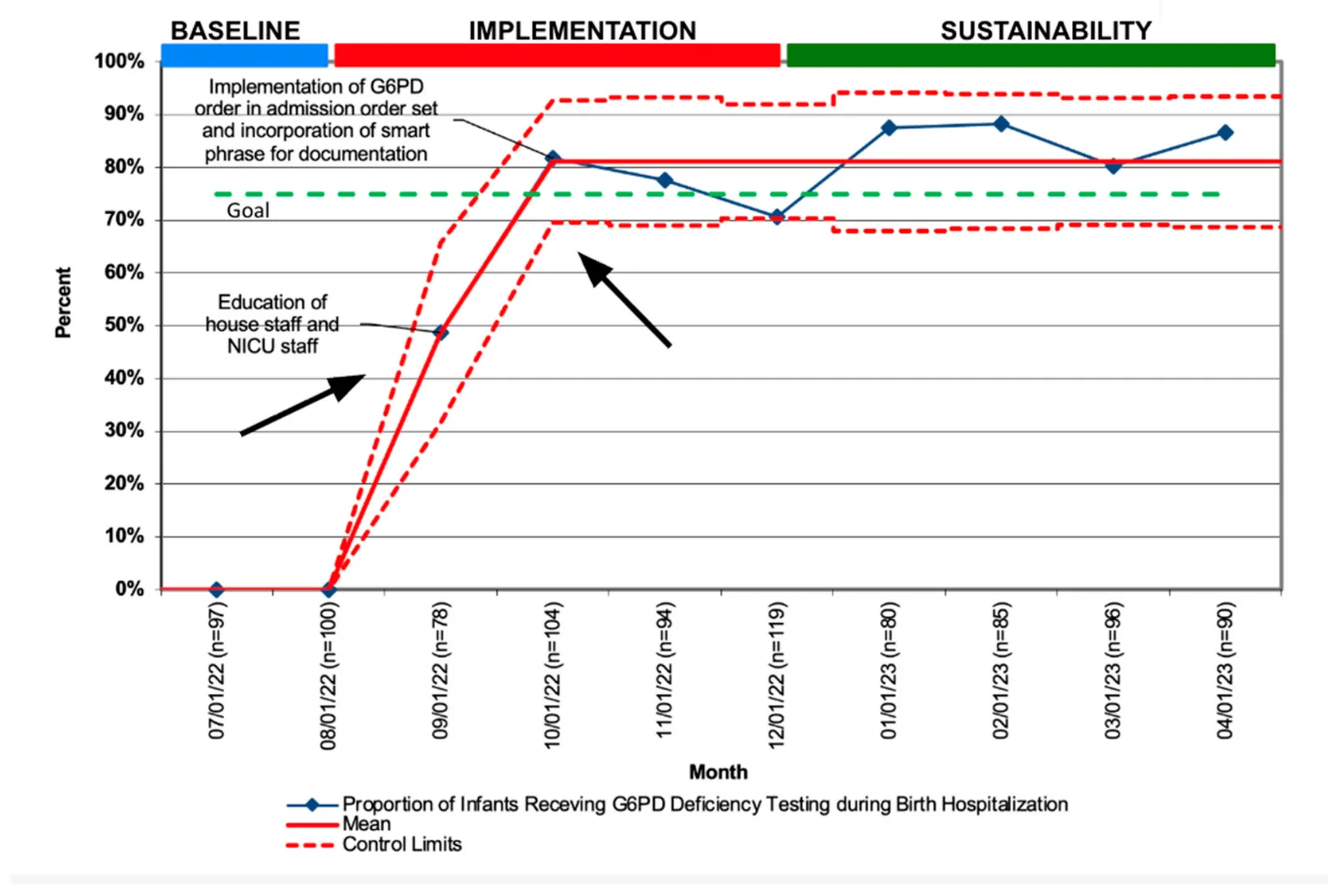
Statistical Process Control Chart with the Proportion of Infants Receiving G6PD Deficiency Testing during Birth Hospitalization. Baseline, intervention, and sustainability periods are indicated on the graph. The two black arrows point to centerline shifts in the SPC chart.

A total of 943 infants were discharged from the well-baby nursery during the study period (July 2022–April 2023): 197 during the July and August 2022 baseline and 746 after the intervention began in September 2022. The flow of infants from discharge through screening, screen-positive identification, referral, and hematology follow-up is summarized in Figure 2. No infants were screened during the July–August 2022 baseline. After staff and provider education began in September 2022 and the quantitative G6PD level was added to the admission order set with a documentation smart-phrase in October 2022, the monthly screening proportion rose sharply and was sustained, with a centerline of 81.1% from October 2022 through April 2023 (monthly range, 70.6–88.2%). This change met criteria for special-cause variation. The initiative exceeded its aim of screening more than 75% of infants within 6 months, and the gain was maintained through the sustainability phase. Figure 3 is the statistical process control chart of the monthly proportion of infants screened for G6PD deficiency. There were no reports of increased workload among nursing or physician staff as a result of the QI initiative.

Part 2: Prevalence of G6PD Deficiency (Observational Component)

**Figure 4:**
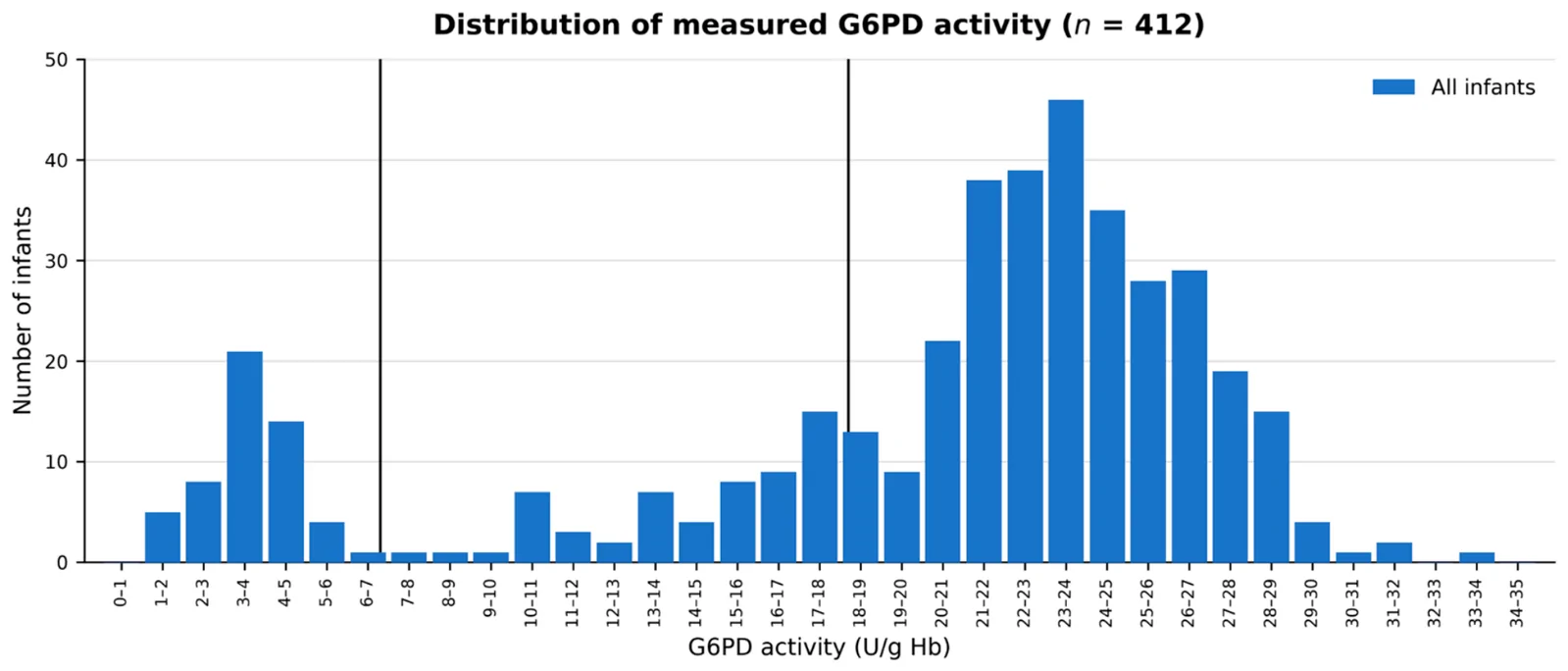
Distribution of measured G6PD activity. Vertical lines in black indicate the population-derived deficiency threshold (6.8 U/g Hb) and the intermediate–normal boundary (18.2 U/g Hb).

**Figure 5:**
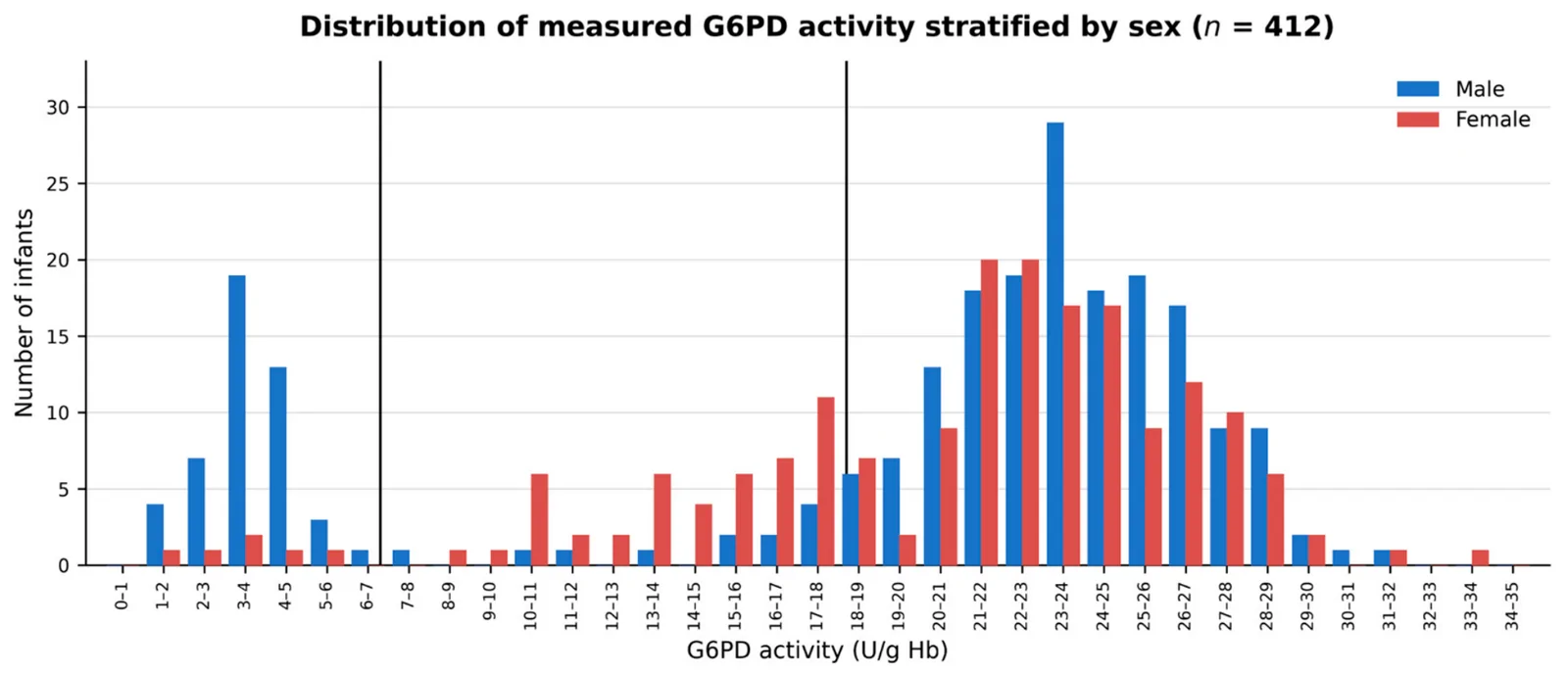
Distribution of measured G6PD activity stratified by sex. Vertical lines in black indicate the population-derived deficiency threshold (6.8 U/g Hb) and the intermediate–normal boundary (18.2 U/g Hb).

A total of 580 infants were screened for G6PD deficiency during the study period. Of 580 screened infants, 52 (9.0%; 95% CI, 6.9–11.6%) screened positive for G6PD deficiency and 87 (15.0%; 95% CI, 12.3–18.1%) had intermediate activity. A numeric G6PD activity value was available for 412 of the 580 screened infants; the remaining 168 (29%) had a normal or high result reported without a numeric value and were retained in prevalence denominators but excluded from distributional analyses. The distribution of measured activity was bimodal (Figure 4). Distribution differed by sex: measured activity among males was bimodal, whereas females showed a unimodal, left-skewed distribution. Male and female distributions are presented separately in Figure 5. The adjusted male median was 22.7 U/g Hb (95% CI, 21.9–23.2), yielding a population-derived deficiency threshold of 6.8 U/g Hb (95% CI, 6.6–7.0) and an intermediate–normal boundary of 18.2 U/g Hb (95% CI, 17.5–18.6). Median activity was 3.7 U/g Hb (IQR, 3.0–4.3) among screen-positive infants, 16.9 (IQR, 13.9–18.3) among those with intermediate activity, and 23.9 (IQR, 22.3–26.0) among normal infants. 14.5% of male infants (47/324; 95% CI, 11.1–18.8%) screened positive compared with 2.0% of female infants (5/256; 95% CI, 0.8–4.5%).

Part 3: Neonatal Outcomes (Observational Component)

The cohort discharged during the study period was predominantly male (54.1%, 510/943) and African American (87.1%, 821/943). The mean birth weight was approximately 3237 g. ABO incompatibility was present in 13.9% (131/943), Rh incompatibility in 3.6% (34/943), and a positive neonatal DAT in 10.3% (97/943) (Table 1). Infants who screened positive for G6PD deficiency were significantly more likely than screen-negative infants to require repeat serum bilirubin testing during the neonatal period, 38.5% (20/52) vs. 19.3% (102/528) (RR 1.99; 95% CI, 1.35–2.93); *p* = 0.002. Screen-positive infants were also more likely to reach a peak serum bilirubin above 10 mg/dL during the birth hospitalization (30.8%, 16/52 vs. 10.2%, 54/528 (RR 3.01; 95% CI, 1.86–4.86); *p* < 0.001, with an even greater difference above 15 mg/dL (19.2%, 10/52 vs. 2.3%, 12/528; RR 8.46; 95% CI, 3.84–18.63); *p* < 0.001. Infants with G6PD deficiency were also more likely to require hospital admission due to hyperbilirubinemia compared to infants without G6PD deficiency, 5.8% (3/52) vs. 0.9% (5/528) (RR 6.09; 95% CI, 1.50–24.77); *p* = 0.028. There were no statistically significant differences between groups in phototherapy during birth hospitalization (0% vs. 2.5%) or the number of ED visits for hyperbilirubinemia (1.9% vs. 0.6%). No infant in either group required IVIG or exchange transfusion (Table 2). Of the 52 screen-positive infants, all were referred to a dedicated pediatric hematology clinic, and 62% (32/52) of the infants attended their pediatric hematology clinics.

## 4. Discussion

Using quality improvement (QI) methodology, we implemented and sustained universal G6PD deficiency screening in a high-risk inner-city well-baby nursery, increasing screening from 0% to 81.1%. Universal screening identified G6PD deficiency in 9% of screened infants. Infants who screened positive for G6PD deficiency were more likely to require repeat serum bilirubin testing during the birth hospitalization; a higher rate of readmission for phototherapy was also observed, although this was based on a few events. Together, these findings demonstrate that a sustainable QI approach can embed universal G6PD screening into routine newborn care in a safety-net population. The largest and most durable improvements followed the incorporation of quantitative G6PD testing into the default admission order set together with a documentation smart-phrase. This suggests that workflow redesign was more effective than education alone in changing clinician behavior. Reusing the blood specimen already obtained for serum bilirubin testing eliminated the need for additional phlebotomy, removing an important feasibility barrier for both clinicians and families. In addition, the program relied primarily on existing resident and nursing workflows rather than new personnel or costly infrastructure, supporting its sustainability. Infants were not screened during the baseline period (July and August 2022), as screening was not yet available. Following implementation, a small proportion of infants remained unscreened. Reasons for non-screening were not routinely documented at the patient level and therefore could not be ascertained by the study team. The screening workflow has remained in place beyond the study period; however, because formal data collection concluded in April 2023, no outcome data were collected thereafter. Consistent with the principles reflected in our key driver framework, the combination of streamlined ordering, standardized documentation, provider education, and integration into routine care created a reliable screening process that could be sustained over time and potentially adapted to similar safety-net settings.

Our findings are generally consistent with the emerging literature on newborn G6PD screening. The 9% screen-positive rate observed in our cohort aligns closely with U.S. prevalence estimates among populations of African ancestry. A large U.S. military study reported an overall prevalence of approximately 9.5% among African Americans [15], and a recent point-of-care study identified a G6PD deficiency rate of 9% of African American pediatric specimens [16]. Given the predominantly African American and African Caribbean population served by our institution, this prevalence is consistent with the expected disease burden. In contrast, Milburn and colleagues reported G6PD deficiency in 4% of newborns during implementation of New York State’s universal screening mandate at an academic center [8]. The higher prevalence of G6PD deficiency in our cohort likely reflects the demographic differences in the populations we serve. The population of African American infants in the Milburn study was 11%, whereas our population was 87% African American. Our study extends this work to a safety-net setting serving a predominantly African American and African Caribbean population, the group most affected by kernicterus disparities and most vulnerable to follow-up gaps, and demonstrates that screening can be embedded and sustained through workflow changes. An important consideration for any screening program is whether the threshold used to define deficiency is appropriate for the population screened. Newborn reference ranges for G6PD activity remain unclear, and cutoffs derived from one population may not transfer to another [8,16,17,18,19]. Rather than rely solely on published thresholds, we derived a deficiency threshold from our own cohort using the adjusted male median, an approach that anchors normality to the population being screened [13]. Because this approach recalculates the reference from the local screened population, the method itself is readily reproducible across laboratories and populations; however, the specific numeric thresholds we report are assay- and population-dependent and should be re-derived locally rather than applied directly elsewhere. Internationally accepted fixed cutoffs were not adopted as the primary definition because newborn reference ranges vary and cutoffs derived from other populations and assays may misclassify infants in our predominantly African-ancestry cohort. The observed sex distribution showed a bimodal pattern among hemizygous males, whereas the female distribution demonstrated a left skew, consistent with the work of Domingo et al., which showed that the female distribution becomes progressively more left-skewed as the prevalence of G6PD deficiency among males rises [20]. We found that screen-positive infants were more likely to undergo repeat serum bilirubin testing during the birth hospitalization. A higher rate of readmission for phototherapy was also observed. These findings are similarly concordant with prior evidence demonstrating a more active bilirubin course among affected newborns. Nock and colleagues reported higher bilirubin levels, greater phototherapy use, and a high prevalence of G6PD deficiency among infants rehospitalized for hyperbilirubinemia [21]. Because repeat bilirubin testing occurred before clinicians were aware of the infant’s G6PD status, this difference is likely to reflect the infant’s clinical course rather than knowledge of the screening result. Screen-positive infants also more frequently reached higher peak serum bilirubin levels, being roughly three times as likely to exceed 10 mg/dL, and eight times as likely to exceed 15 mg/dL. Because peak bilirubin is an objective, threshold-based measure rather than a clinician-initiated action, this finding is less susceptible to surveillance bias than repeat serum bilirubin testing and provides more direct evidence of a greater hyperbilirubinemia burden among screen-positive infants. This aligns with the existing literature: infants with G6PD deficiency are more likely to have hyperbilirubinemia and higher peak bilirubin levels [22]. Although we did not observe differences in phototherapy during birth hospitalization, intravenous immunoglobulin use, or exchange transfusion, these outcomes were uncommon, and the number of screen-positive infants was relatively small. This also aligns with the findings of Dalldorf et al., where infants with G6PD deficiency were more likely to require phototherapy, but exchange transfusions and bilirubin-induced neurotoxicity were rare [23].

The broader significance of this work lies in its implications for health equity. G6PD deficiency is a major contributor to racial disparities in severe neonatal hyperbilirubinemia and kernicterus [24]. African American infants have a lower incidence of hyperbilirubinemia; however, they account for 25% of all reported kernicterus cases in the US. G6PD accounts for around 60% of these cases [25]. The 2009 clinical report from the Pilot USA Kernicterus Registry, a landmark root-cause analysis of 125 infants with kernicterus, found that 20% of the infants who developed kernicterus had G6PD deficiency. The major root cause identified was systems failure at multiple sites and by multiple healthcare providers and an inability to identify at-risk infants [26]. Paradoxically, the New York State mandate that prompted programs such as ours relies on risk factors that include racial and ethnic ancestry. Such an approach risks perpetuating disparities and does not reliably identify genetic risk [27]. Moreover, G6PD deficiency occurs in populations traditionally considered lower risk. Investigators have estimated that risk-factor-based screening would miss approximately 44% of affected newborns, including infants whose parents identify as White or Ashkenazi Jewish and as Puerto Rican or Dominican [27,28]. This concern is consistent with the broader movement away from race-based clinical decision-making reflected in the 2022 AAP hyperbilirubinemia guideline, which removed race as a modifier of risk for hyperbilirubinemia [4,29]. Universal pre-discharge screening has therefore been proposed as the most equitable detection strategy [30], and economic analyses suggest that universal screening is cost-effective [31]. In this context, our decision to implement universal rather than risk-factor-based screening removes clinician race-based judgment from the testing decision and ensures that all infants in a high-prevalence, follow-up-vulnerable population receive screening. The strengths of this initiative include its implementation in a real-world, safety-net setting serving a large, predominantly African American and African Caribbean population at disproportionate risk for severe hyperbilirubinemia and kernicterus. The successful integration of universal screening into existing resident- and nurse-driven workflows without requiring additional phlebotomy represents an additional strength. Importantly, the derivation of population-specific G6PD activity thresholds in this population adds to the limited literature on context-specific thresholds for G6PD deficiency screening.

Our study must be interpreted in the setting of several limitations. Outcome comparisons were limited by the relatively small number of screen-positive infants and the rarity of severe clinical events. As a result, analyses of phototherapy, intravenous immunoglobulin use, and exchange transfusion were not powered a priori. Larger cohorts may detect differences that were not apparent in this study. In addition, outcome comparisons were unadjusted for gestational age, birth weight, sex, direct antiglobulin test positivity, Rh incompatibility, and ABO incompatibility, so residual confounding cannot be excluded. Due to the small number of screen-positive infants and the rarity of severe outcomes, multivariable adjustment for these variables was not statistically feasible; the reported comparisons should therefore be interpreted as unadjusted associations. Importantly, the difference in hospital readmission for phototherapy was based on only 3 of 52 infants in the screen-positive group and 5 of 528 infants in the screen-negative group. Despite reaching statistical significance, these results must be interpreted with caution because the event numbers are very small. They should be regarded as hypothesis-generating and do not constitute sufficient evidence to establish an association.

The study was conducted at a single institution serving a high-prevalence, predominantly African-ancestry population, and the observed 9% screen-positive rate and locally derived thresholds may not transfer directly to hospitals serving lower-prevalence or more heterogeneous populations. This initiative was conducted within a resident- and nurse-driven workflow, and implementation may differ in nurseries with alternative staffing structures. Data regarding cancelled testing or levels not sent due to parental refusal were not collected. Data regarding the average turnaround time for results to be available was also not collected. Because specimens were processed by an external reference laboratory, results were frequently unavailable until after discharge, shifting confirmatory testing and counseling responsibilities to outpatient providers and creating the potential for incomplete follow-up, particularly among infants receiving care outside our health system. The underlying approach of embedding universal screening into routine admission orders and reusing the bilirubin specimen is broadly applicable, and the adjusted-male-median method allows each site to establish locally valid thresholds. Only 62% of screen-positive infants attended the dedicated hematology clinic. The reason for missed appointments was not available to the study team. Incomplete follow-up may lead to underascertainment of confirmed diagnoses and downstream outcomes. Emerging quantitative point-of-care assays may mitigate this limitation by enabling pre-discharge result availability [31]. The quantitative enzyme assay functions as a screening rather than a diagnostic test. Enzyme activity may appear falsely normal during or shortly after hemolysis. Newborn-specific reference ranges for defining deficiency continue to evolve [8], and our study period included implementation and early sustainability phases only. Longer-term sustainability, readmission outcomes, and neurodevelopmental outcomes were beyond the scope of this project. Lastly, 29% of screened infants had a normal or high result reported without a numeric value. Because these were, by definition, the highest values in the distribution, the upper portion of the reference range could not be characterized and we do not report an upper reference limit; the deficiency threshold, which depends on the lower tail of the distribution, was unaffected.

## 5. Conclusions

Quality improvement methods can reliably and sustainably integrate universal G6PD deficiency screening into routine newborn care in a high-risk safety-net nursery without additional phlebotomy. The high proportion of infants screening positive for G6PD deficiency and the increased need for repeat bilirubin testing among these infants underscore the clinical relevance of screening in this population. Universal screening offers a practical and equity-focused alternative to race- and ancestry-based testing strategies and may support efforts to reduce disparities in severe hyperbilirubinemia and kernicterus. However, since this was primarily a quality improvement initiative and the clinical outcome analyses were observational, the study was not designed to demonstrate such reductions. Future work should focus on reducing turnaround time through point-of-care testing, expanding screening to additional newborn settings, including the neonatal intensive care unit, strengthening systems for confirmatory testing and follow-up, and evaluating longer-term clinical outcomes in larger cohorts.

## Figures and Tables

**Table 1:** Characteristics of all infants discharged from the well-baby nursery during the study period.

Characteristic	All Infants (*n* = 943)
Sex	
Male	510 (54.1%)
Female	433 (45.9%)
Gestational age	
Median, wk	39
34–36 wk	32 (3.4%)
37–38 wk	327 (34.7%)
39–40 wk	534 (56.6%)
41–43 wk	50 (5.3%)
Mean birth weight, g	3237
Maternal race/ethnicity	
African American	821 (87.1%)
Other	87 (9.2%)
Asian	16 (1.7%)
White	10 (1.1%)
Unknown	8 (0.8%)
Native American	1 (0.1%)
Neonatal blood group	
O	476 (50.5%)
A	263 (27.9%)
B	170 (18%)
AB	34 (3.6%)
Rh positive	887 (94.1%)
Rh negative	56 (5.9%)
Hemolysis risk factors	
ABO incompatibility	131 (13.9%)
Rh incompatibility	34 (3.6%)
Positive maternal antibody screen	51 (5.4%)
Positive neonatal DAT (Coombs)	97 (10.3%)
G6PD screening status	
Screened	580 (61.5%)
Deficient (low) among screened infants	52 (9%)
Not screened	363 (38.5%)

Table 1 footnote. Data are *n* (%) of all infants discharged from the well-baby nursery (*n* = 943). DAT—direct antiglobulin test; G6PD—glucose-6-phosphate dehydrogenase; Rh—Rhesus.

**Table 2:** Early neonatal outcomes among screened infants who screened positive and negative for G6PD deficiency.

	Screen Positive for G6PD Deficiency (*n* = 52)	Screen Negative for G6PD Deficiency (*n* = 528)	*p* Value
Repeat serum bilirubin testing	20 (38.5%)	102 (19.3%)	0.002
Peak serum bilirubin >10 mg/dL	16 (30.8%)	54 (10.2%)	<0.001
Peak serum bilirubin >15 mg/dL	10 (19.2%)	12 (2.3%)	<0.001
Phototherapy during birth hospitalization	0 (0.0%)	13 (2.5%)	0.62
Hospitalization for hyperbilirubinemia	3 (5.8%)	5 (0.9%)	0.028
Emergency department visit	1 (1.9%)	3 (0.6%)	0.31
IVIG	0 (0.0%)	0 (0.0%)	NE
Exchange transfusion	0 (0.0%)	0 (0.0%)	NE

Table 2 footnote. Data are *n* (%). *p* values were calculated using the Fisher exact test. Relative risks (RR) with 95% confidence intervals for the key outcomes are reported in the text. G6PD—glucose-6-phosphate dehydrogenase; IVIG—intravenous immunoglobulin; NE—not estimable (no events in either group).

## Data Availability

The data presented in this study are available on request from the corresponding author.
